# Synthesis and structure of (*Z*)-8-methyl-2-(8-methyl-2,3,4,9-tetra­hydro­carbazol-1-yl­idene)-2,3,4,9-tetra­hydro­carbazol-1-one

**DOI:** 10.1107/S2056989026003427

**Published:** 2026-04-10

**Authors:** Makuteswaran Sridharan, Aravazhi Amalan Thiruvalluvar

**Affiliations:** a Department of Chemistry, RV College of Engineering, Bangalore 560 059, Karnataka, India; b Principal (Retired), 63 Shanthi Nagar, 5th Street, Nanjikottai Road, Thanjavur 613 006, Tamilnadu, India; University of Aberdeen, United Kingdom

**Keywords:** crystal structure, dicarbazole, Hirshfeld surface, N—H⋯O hydrogen bonding, C—H⋯π and π–π contacts

## Abstract

In the title compound, C_26_H_24_N_2_O, the dihedral angle between the indole fused ring units is 36.37 (5)° and an intra­molecular N—H⋯O hydrogen bond closes an *S*(7) ring. In the extended structure, inversion dimers linked by pairwise N—H⋯O hydrogen bonds generate an *R*^2^_2_(10) loop. Secondary C—H⋯π contacts consolidate the packing and a π–π stacking inter­action is also observed. The contributions of the different inter­actions towards the crystal packing were analysed using Hirshfeld surface and fingerprint plots, showing that the largest contributions come from H⋯H (59.5%) and C⋯H/H⋯C contacts (28.5%).

## Abstract 2

In the title compound, the dihedral angle between the indole fused ring units is 36.37 (5)° and an intra­molecular N—H⋯O hydrogen bond closes an *S*(7) ring. In the extended structure, inversion dimers linked by pairwise N—H⋯O hydrogen bonds generate an *R*^2^_2_(10) loop. Secondary C—H⋯π contacts consolidate the packing and a π–π stacking inter­action is also observed.

## 1. Chemical context

Dicarbazole derivatives have attracted considerable inter­est in organic optoelectronics due to their high hole mobility, excellent thermal stability, and robust electrochemical durability (Matsuda *et al.*, 2025 ▸). Synthetic strategies for these compounds often involve cascade annulations, palladium-catalysed tandem reactions, or oxidative cyclizations, enabling access to highly functionalized frameworks. With this view, an attempt has been invested to prepare these classes of compounds using 2,3,4,9-tetra­hydro­carbazol-1-ones (Sridharan *et al.*, 2026 ▸) as precursors *via* an easily accessible inter­mediate. As part of these studies, we now describe the synthesis, crystal structure and Hirshfeld surface analysis of the title compound, C_26_H_24_N_2_O (**I**).
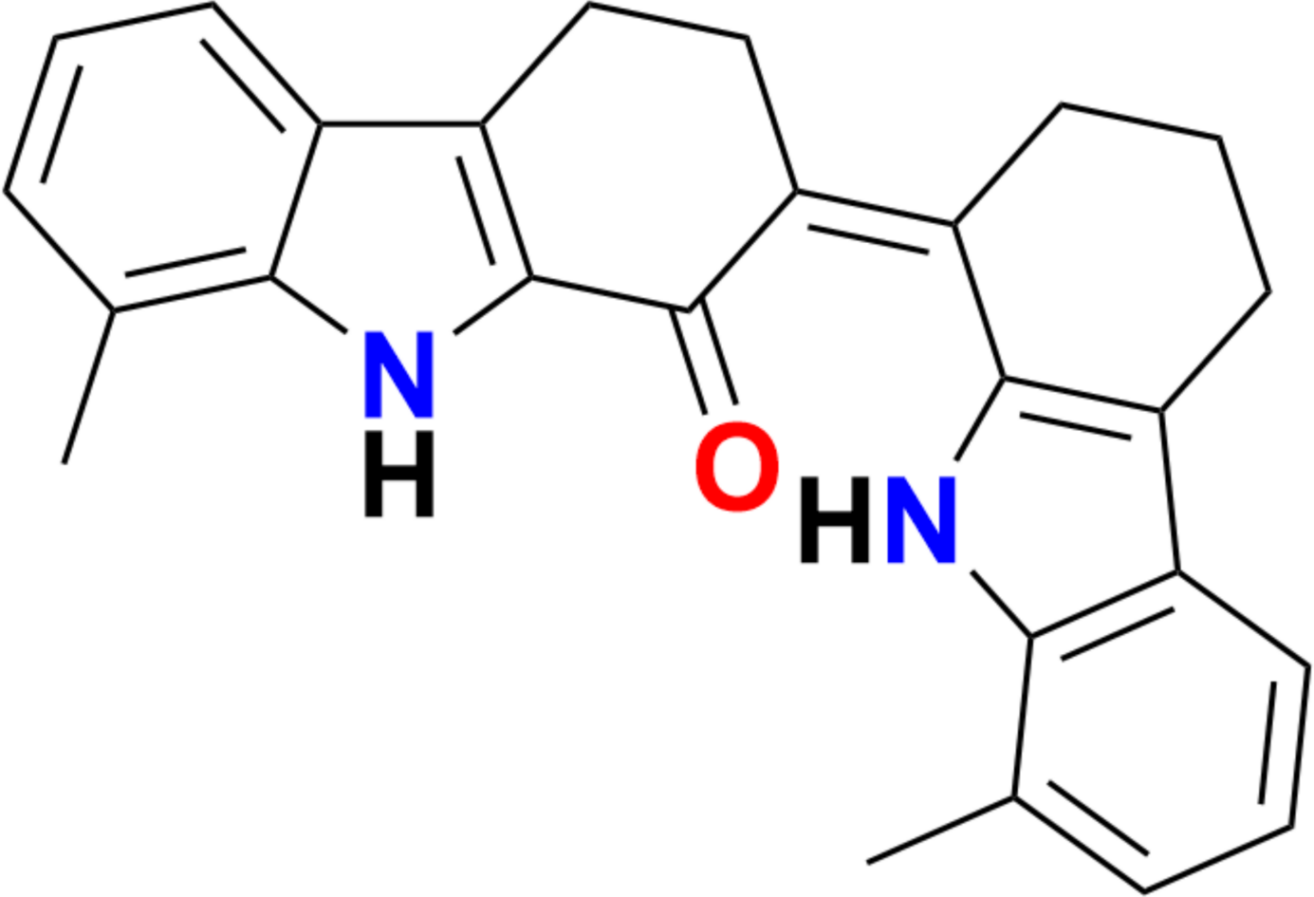


## 2. Structural commentary

**Figure 1:**
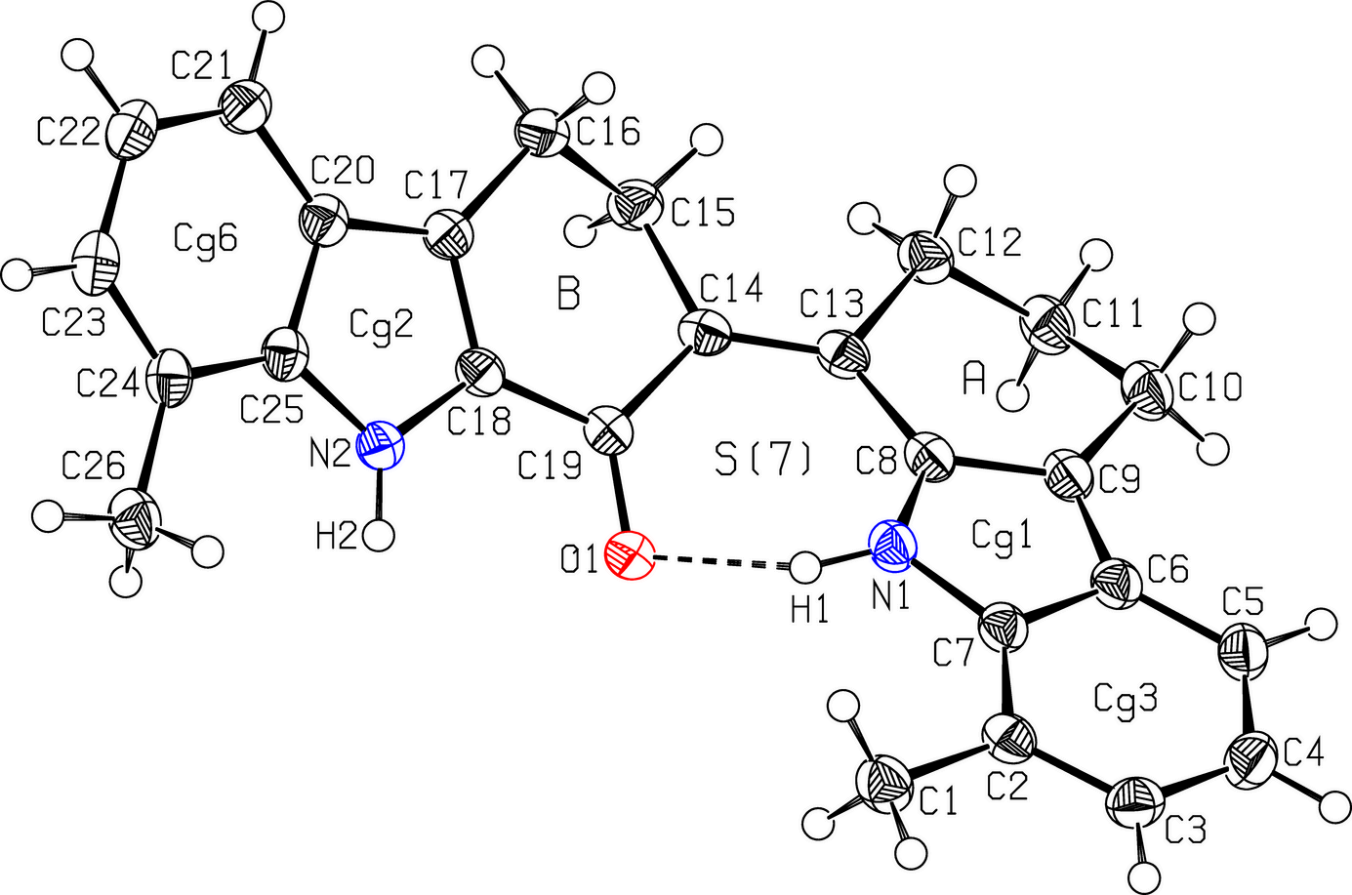
The mol­ecular structure of (**I**), showing displacement ellipsoids drawn at the 50% probability level.

As shown in Fig. 1 ▸, compound (**I**) consists of two indole and two cyclo­hexene units fused *via* the C13—C14 bond. The dihedral angle between the pyrrole rings (C6–C9/N1 and C18/C17/C20/C25/N2) is 37.29 (8)°. The first pair of fused pyrrole and benzene (C2–C7) rings are nearly co-planar, subtending a dihedral angle of 3.13 (8)°. Similarly, the dihedral angle between the second pair of pyrrole and benzene (C20–C25) rings is 3.02 (7)°. The dihedral angle between the benzene rings is 35.99 (7)°.

A puckering analysis (Cremer & Pople, 1975 ▸) of the six-membered *A* (C8–C13) cyclo­hexene ring gave the parameters: *q*_2_ = 0.3516 (16) Å, *q*_3_ = −0.2825 (16) Å, *Q*_T_ = 0.4510 (16) Å, θ = 128.8 (2)° and φ = 12.2 (3)°, corresponding to an envelope conformation where atom C11 is at the flap position and displaced by 0.615 (2) Å from best plane of the remaining atoms. A similar analysis for ring *B* (C14–C19) gave *q*_2_ = 0.3925 (15) Å, *q*_3_ = −0.2247 (15) Å, *Q*_T_ = 0.4523 (15) Å, θ = 119.79 (19)° and φ = 240.7 (2)°, indicating an envelope conformation, where atom C15 is at the flap position and 0.621 (2) Å away from best plane of the remaining atoms. An intra­molecular N1—H1⋯O1 hydrogen bond forms an *S*(7) ring motif (Fig. 1 ▸ and Table 1 ▸).

## 3. Supra­molecular features

**Figure 2:**
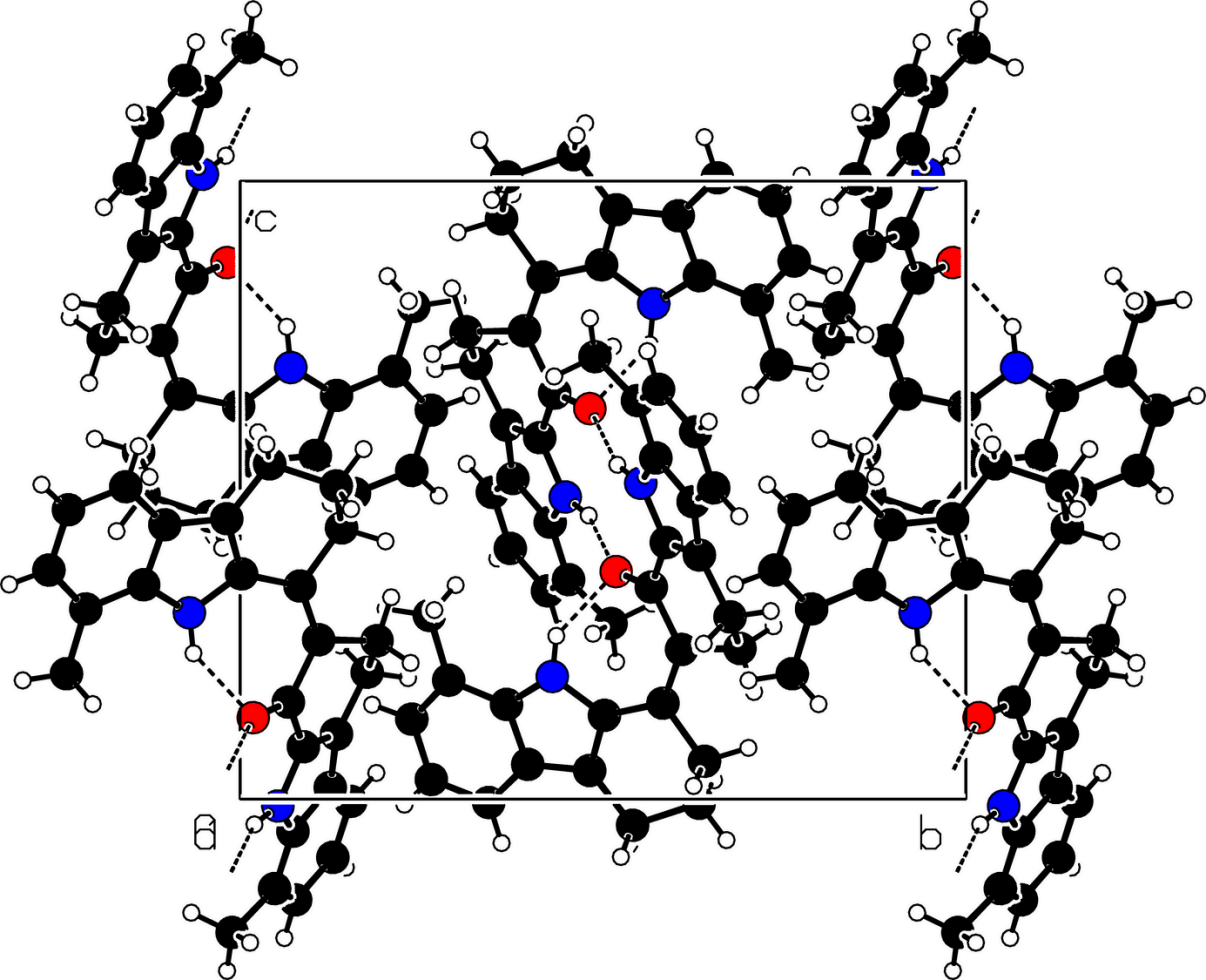
Partial packing view of (**I**), viewed down the *a-*axis direction with black dashed lines representing N—H⋯O hydrogen bonds.

**Figure 3:**
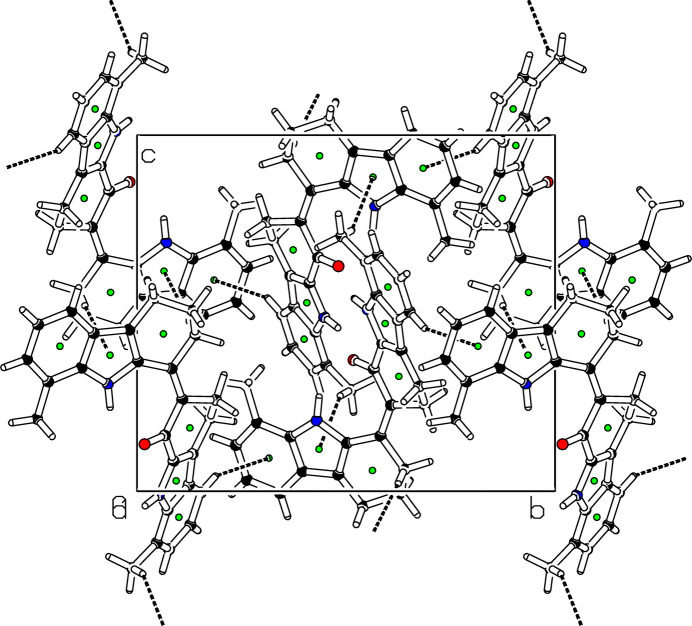
Straw-style packing view of (**I**), viewed down the *a-*axis direction, showing the C—H⋯π contacts. Centroids are given as green spheres and black dashed lines are H⋯π contacts.

**Figure 4:**
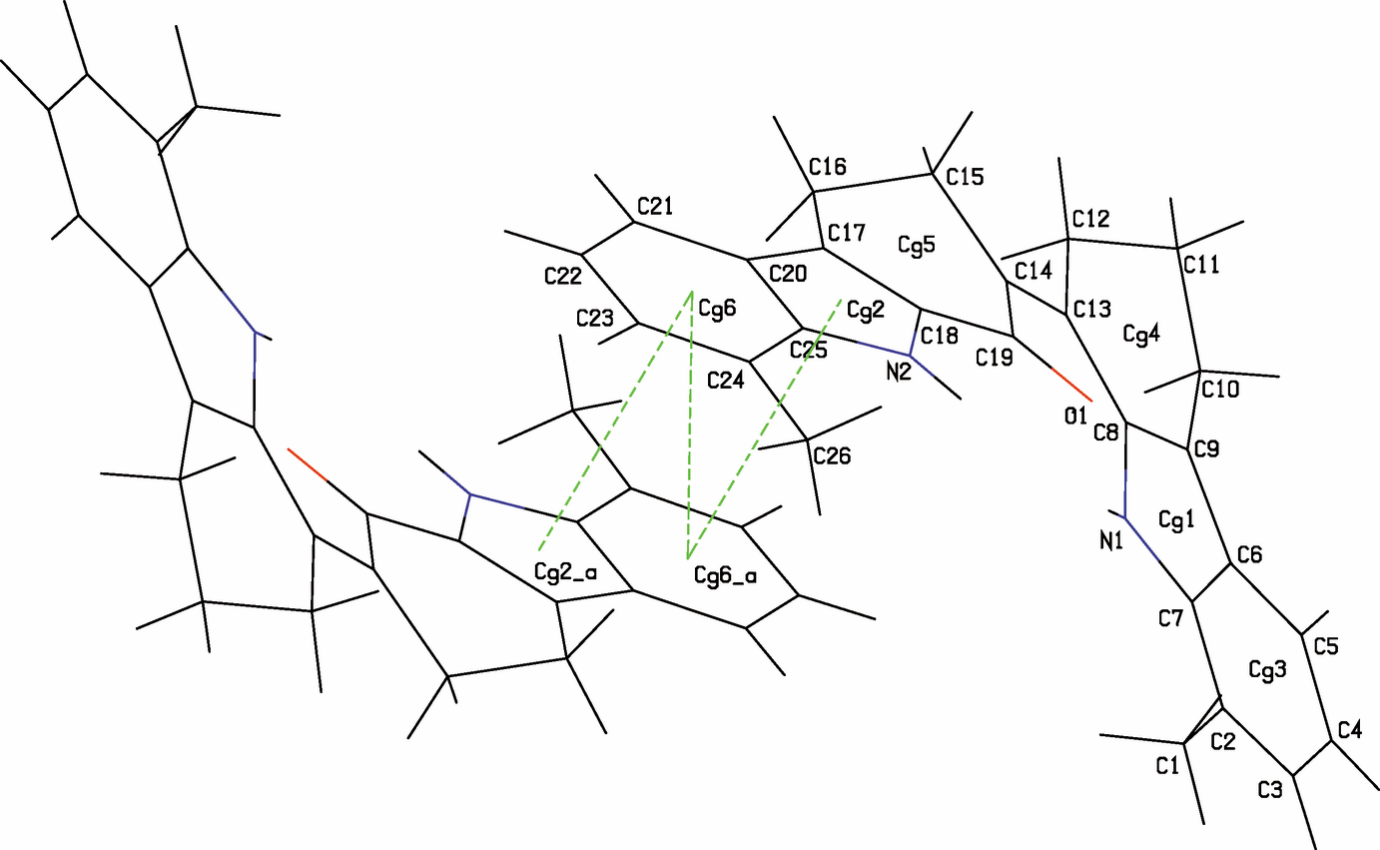
The stick-style crystal structure of (**I**), showing the formation of π–π stacking inter­actions [Symmetry code: (*a*) 1 − *x*, −*y*, −*z*]. Green dashed lines indicate the π–π contacts.

In the crystal, the mol­ecules of (**I**) associate *via* pairwise N2—H2⋯O1^i^ [symmetry code: (i) 2 − *x*, −*y*, −*z*] hydrogen bonds (Table 1 ▸) into inversion dimers with an 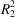
(10) loop graph-set motif (Fig. 2 ▸). The packing also exhibits three C—H⋯π inter­actions (Fig. 3 ▸ and Table 1 ▸) involving the pyrrole (N1/C7/C6/C9/C8) and the benzene (C2–C7) rings. The mol­ecules further exhibit slipped π–π stacking inter­actions: *Cg*2⋯*Cg*6(1 − *x*, −*y*, −*z*) = 3.5739 (15) Å, slippage = 0.891 Å and *Cg*6⋯*Cg*6(1 − *x*, −*y*, −*z*) = 3.6763 (16) Å, slippage = 1.246 Å; where *Cg*2 and *Cg*6 are the centroids of the pyrrole ring (N2/C18/C17/C20/C25) and the benzene ring (C20–C25) respectively (Fig. 4 ▸).

## 4. Database survey

A search of the Cambridge Structural Database (CSD, Version 6.01, updated to November 2025; Groom *et al.*, 2016 ▸) using the core structure of (**I**) gave zero hits.

## 5. Hirshfeld surface (HS) and 2D fingerprint plots

**Figure 5:**
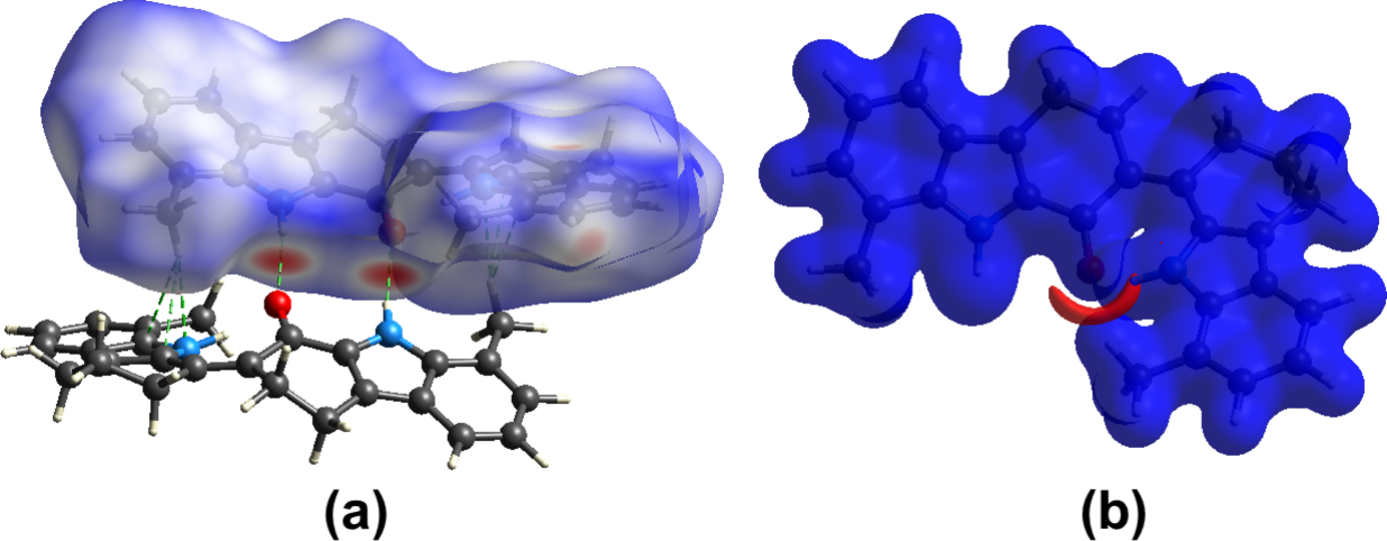
(*a*) View of the three-dimensional Hirshfeld surface of (**I**), plotted over *d*_norm_ in the range from −0.48 to 1.34 a.u. with a neighbouring mol­ecule. The inter­molecular hydrogen bonds are depicted by green dashed lines. (*b*) View of the three-dimensional electrostatic potential surface of (**I**) plotted over the range from −0.05 to 0.05 a.u., using the STO-3 G basis set at the Hartree–Fock method of theory.

*CrystalExplorer* (Version 21.5; Spackman *et al.*, 2021 ▸) was used to investigate and visualize further the inter­molecular inter­actions of (**I**). The HS plotted over *d*_norm_ in the range from −0.48 to 1.34 a.u. is shown in Fig. 5 ▸(*a*). The electrostatic potential surface using the STO-3G basis set at the Hartree Fock level of theory and mapped on the Hirshfeld surface over the range from −0.05 to 0.05 a.u. clearly shows the positions of the close inter­molecular contacts in the compound [Fig. 5 ▸(*b*)]. The positive electrostatic potential (blue area) over the surface indicates hydrogen-donor potential, whereas the negative (red area) represents the hydrogen-bond acceptors.

**Figure 6:**
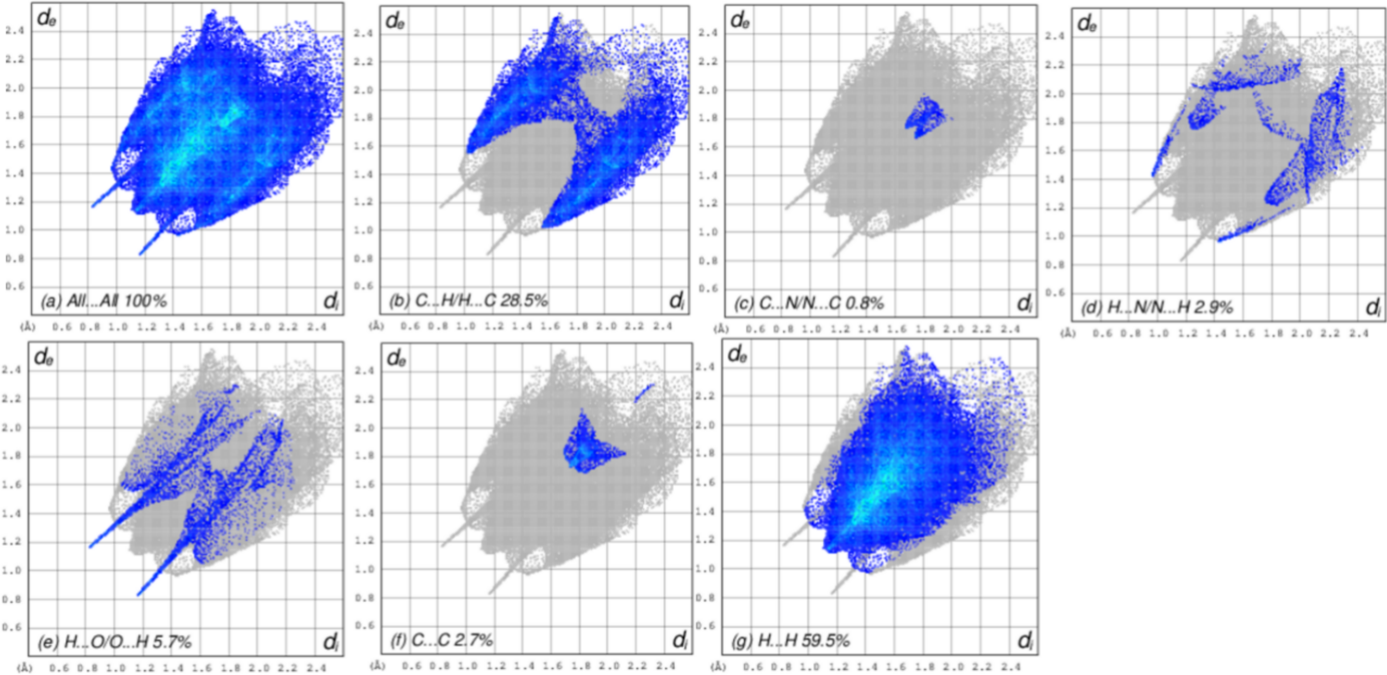
Two-dimensional fingerprint plots for (**I**), showing (*a*) all inter­actions, and delineated into (*b*) C⋯H/H.·C, (*c*) C⋯N/N⋯C, (*d*) H⋯N/N⋯H, (*e*) H⋯O/O⋯H, (*f*) C⋯C and (g*)* H⋯H inter­actions. The *d*_i_ and *d*_e_ values are the closest inter­nal and external distances (in Å) from given points on the Hirshfeld surface.

The overall two-dimensional fingerprint plot is shown in Fig. 6 ▸(*a*), while those delineated into C⋯H/H⋯C, C⋯N/N⋯C, H⋯N/N⋯H, H⋯O/O⋯H, C⋯C and H⋯H contacts are illustrated in Fig. 6 ▸(*b*)–6(*g*), respectively, together with their relative contributions to the Hirshfeld surface. The most significant inter­action type is H⋯H, contributing 59.5% to the Hirshfeld surface, which is reflected in Fig. 6 ▸(*g*) as widely scattered points of high density due to the large hydrogen content of the mol­ecule. In the presence of C⋯H inter­actions, the pair of characteristic wings in the fingerprint plot is delineated into C⋯H/H⋯C contacts [28.5% contribution to the HS; Fig. 6 ▸(*b*)]. The C⋯N/N⋯C contacts contribute only 0.8% [Fig. 6 ▸(*c*)]. The H⋯N/N⋯H contacts contribute 2.9% [Fig. 6 ▸(*d*)]. The H⋯O/O⋯H contribute 5.7% [Fig. 6 ▸(*e*)] and finally, the C⋯C contacts [Fig. 6 ▸(*f*)] contribute only 2.7%. The packing of (**I**) is thus dominated by van der Waals inter­actions despite the presence of N—H⋯O hydrogen bonds.

## 6. Synthesis and crystallization

**Figure 7:**
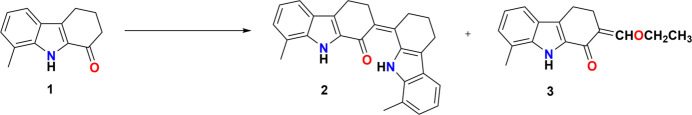
The synthesis scheme for (**I**).

8-Methyl-2,3,4,9-tetrahydrocarbazol-1-one (1.0 g, 0.005 mol) in di­chloro­methane (15 ml) was added to an ice-cooled solution of di­eth­oxy­carbenium fluoro­borate (prepared *in situ* from BF_3_·Et_2_O (1.65 ml, 0.01 mol) and HC(OEt_3_) (1.25 ml, 0.01 mol). The reaction mixture was kept at 258–263 K. To this mixture, tri­ethyl­amine (0.01 mol) was added dropwise and the stirring was continued over a period of five h. The reaction was monitored by TLC. After the completion of the reaction, the excess solvent was then removed and extracted using ethyl acetate dried over anhydrous sodium sulfate. The brown solid separated out was then separated by column chromatography over silica gel using petroleum ether: ethyl acetate as eluants (99:1) and (95:5) to yield (*Z*)-8-methyl-2,3,4,9-tetra­hydro-2-(8′-methyl-2′,3′,4′,9′-tetra­hydro­carbazol-1-yl­idene)-carbazol-1-one (**2**) and (*Z*)-2-(eth­oxy­methyl­ene)-8-methyl-2,3,4,9-tetra­hydro-1*H*-carbazol-1-one (**3**), respectively. The chemical structure of the final products was confirmed by NMR Spectroscopy and elemental analysis data. Compound **2** was recrystallized using ethanol as solvent as yellow prisms of (**I**) (0.355 g, 18%), m.p.415–417 K. The rection scheme is shown in Fig. 7 ▸.

## 7. Refinement

Crystal data, data collection and structure refinement details are summarized in Table 2 ▸. The N-bonded H atoms were located in a difference Fourier map and refined isotropically with *U*_iso_(H) = 1.2*U*_eq_(N). All the other H atoms were placed in calculated positions and were refined as riding atoms with *U*_iso_(H) = 1.2*U*_eq_(C) or 1.5*U*_eq_(methyl C). The methyl hydrogen atoms were allowed to rotate, but not to tip, to best fit the experimental electron density.

## Supplementary Material

Crystal structure: contains datablock(s) I. DOI: 10.1107/S2056989026003427/hb8200sup1.cif

Structure factors: contains datablock(s) I. DOI: 10.1107/S2056989026003427/hb8200Isup2.hkl

Supporting information file. DOI: 10.1107/S2056989026003427/hb8200Isup3.cdx

Supporting information file. DOI: 10.1107/S2056989026003427/hb8200Isup4.cml

CCDC reference: 1540676

Additional supporting information:  crystallographic information; 3D view; checkCIF report

## Figures and Tables

**Table 1:** Hydrogen-bond geometry (Å, °) *Cg*1 and *Cg*3 are the centroids of the pyrrole (N1/C7/C6/C9/C8) and benzene (C2–C7) rings, respectively.

*D*—H⋯*A*	*D*—H	H⋯*A*	*D*⋯*A*	*D*—H⋯*A*
N1—H1⋯O1	0.887 (18)	1.887 (18)	2.6470 (16)	142.6 (15)
N2—H2⋯O1^i^	0.887 (19)	2.103 (19)	2.9574 (17)	161.4 (16)
C12—H12*B*⋯*Cg*1^ii^	0.99	2.90	3.876 (2)	170
C21—H21⋯*Cg*3^iii^	0.95	2.86	3.736 (2)	154
C26—H26*C*⋯*Cg*1^i^	0.98	2.70	3.549 (2)	145

Symmetry codes: (i) 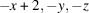; (ii) 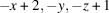; (iii) 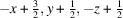.

**Table 2:** Experimental details

Crystal data	
Chemical formula	C_26_H_24_N_2_O
*M*_r_	380.47
Crystal system, space group	Monoclinic, *P*2_1_/*n*
Temperature (K)	100
*a*, *b*, *c* (Å)	9.424 (3), 15.566 (5), 13.530 (5)
β (°)	101.748 (6)
*V* (Å^3^)	1943.3 (11)
*Z*	4
Radiation type	Mo *K*α
μ (mm^−1^)	0.08
Crystal size (mm)	0.55 × 0.45 × 0.35

Data collection	
Diffractometer	Bruker SMART APEX CCD
Absorption correction	Multi-scan (*SADABS*; Krause *et al.*, 2015 ▸)
*T*_min_, *T*_max_	0.907, 0.973
No. of measured, independent and observed [*I* > 2σ(*I*)] reflections	15853, 4766, 3926
*R*_int_	0.030
(sin θ/λ)_max_ (Å^−1^)	0.667

Refinement	
*R*[*F*^2^ > 2σ(*F*^2^)], *wR*(*F*^2^), *S*	0.050, 0.130, 1.07
No. of reflections	4766
No. of parameters	270
H-atom treatment	H atoms treated by a mixture of independent and constrained refinement
Δρ_max_, Δρ_min_ (e Å^−3^)	0.32, −0.22

Computer programs: *SMART* (Bruker, 2002 ▸), *SAINT-Plus* (Bruker, 2003 ▸), *SHELXS* (Sheldrick, 2008 ▸), *SHELXL2025/1* (Sheldrick, 2015 ▸), *ORTEP-3 for Windows* (Farrugia, 2012 ▸), *PLATON* (Spek, 2020 ▸) and *publCIF* (Westrip, 2010 ▸).

## supplementary crystallographic information

### (Z)-8-Methyl-2-(8-methyl-2,3,4,9-tetrahydrocarbazol-1-ylidene)-2,3,4,9-tetrahydrocarbazol-1-one Crystal data

**Table:**

C_26_H_24_N_2_O	*F*(000) = 808
*M**_r_* = 380.47	*D*_x_ = 1.300 Mg m^−^^3^
Monoclinic, *P*2_1_/*n*	Mo *K*α radiation, λ = 0.71073 Å
*a* = 9.424 (3) Å	Cell parameters from 7497 reflections
*b* = 15.566 (5) Å	θ = 2.4–30.5°
*c* = 13.530 (5) Å	µ = 0.08 mm^−^^1^
β = 101.748 (6)°	*T* = 100 K
*V* = 1943.3 (11) Å^3^	Block, red
*Z* = 4	0.55 × 0.45 × 0.35 mm

### (Z)-8-Methyl-2-(8-methyl-2,3,4,9-tetrahydrocarbazol-1-ylidene)-2,3,4,9-tetrahydrocarbazol-1-one Data collection

**Table:**

Bruker SMART APEX CCD diffractometer	4766 independent reflections
Radiation source: fine-focus sealed tube	3926 reflections with *I* > 2σ(*I*)
Graphite monochromator	*R*_int_ = 0.030
ω scans	θ_max_ = 28.3°, θ_min_ = 2.0°
Absorption correction: multi-scan (SADABS; Krause *et al.*, 2015)	*h* = −10→12
*T*_min_ = 0.907, *T*_max_ = 0.973	*k* = −20→20
15853 measured reflections	*l* = −18→18

### (Z)-8-Methyl-2-(8-methyl-2,3,4,9-tetrahydrocarbazol-1-ylidene)-2,3,4,9-tetrahydrocarbazol-1-one Refinement

**Table:**

Refinement on *F*^2^	Primary atom site location: structure-invariant direct methods
Least-squares matrix: full	Secondary atom site location: difference Fourier map
*R*[*F*^2^ > 2σ(*F*^2^)] = 0.050	Hydrogen site location: mixed
*wR*(*F*^2^) = 0.130	H atoms treated by a mixture of independent and constrained refinement
*S* = 1.07	*w* = 1/[σ^2^(*F*_o_^2^) + (0.0626*P*)^2^ + 0.5658*P*] where *P* = (*F*_o_^2^ + 2*F*_c_^2^)/3
4766 reflections	(Δ/σ)_max_ < 0.001
270 parameters	Δρ_max_ = 0.32 e Å^−^^3^
0 restraints	Δρ_min_ = −0.21 e Å^−^^3^

### (Z)-8-Methyl-2-(8-methyl-2,3,4,9-tetrahydrocarbazol-1-ylidene)-2,3,4,9-tetrahydrocarbazol-1-one Special details

**Table:**

Geometry. All esds (except the esd in the dihedral angle between two l.s. planes) are estimated using the full covariance matrix. The cell esds are taken into account individually in the estimation of esds in distances, angles and torsion angles; correlations between esds in cell parameters are only used when they are defined by crystal symmetry. An approximate (isotropic) treatment of cell esds is used for estimating esds involving l.s. planes.

### (Z)-8-Methyl-2-(8-methyl-2,3,4,9-tetrahydrocarbazol-1-ylidene)-2,3,4,9-tetrahydrocarbazol-1-one Fractional atomic coordinates and isotropic or equivalent isotropic displacement parameters (Å^2^)

**Table:**

	*x*	*y*	*z*	*U*_iso_*/*U*_eq_	
C1	1.15744 (18)	−0.23918 (10)	0.20283 (11)	0.0336 (3)	
H1A	1.192886	−0.203061	0.153681	0.050*	
H1B	1.052243	−0.232395	0.194170	0.050*	
H1C	1.180339	−0.299449	0.192142	0.050*	
C2	1.22915 (16)	−0.21259 (9)	0.30769 (10)	0.0275 (3)	
C3	1.32978 (16)	−0.26340 (10)	0.37065 (11)	0.0312 (3)	
H3	1.352957	−0.318172	0.347315	0.037*	
C4	1.39898 (17)	−0.23656 (11)	0.46818 (11)	0.0341 (3)	
H4	1.466906	−0.273631	0.508888	0.041*	
C5	1.36983 (16)	−0.15755 (10)	0.50546 (11)	0.0314 (3)	
H5	1.416963	−0.139813	0.571141	0.038*	
C6	1.26840 (15)	−0.10365 (9)	0.44384 (10)	0.0270 (3)	
C7	1.19930 (15)	−0.13284 (9)	0.34684 (10)	0.0251 (3)	
C8	1.12132 (15)	0.00201 (9)	0.36441 (10)	0.0250 (3)	
C9	1.21708 (15)	−0.01840 (9)	0.45374 (10)	0.0268 (3)	
C10	1.25226 (17)	0.04076 (10)	0.54245 (10)	0.0319 (3)	
H10A	1.356465	0.036316	0.573907	0.038*	
H10B	1.195250	0.024603	0.593506	0.038*	
C11	1.21602 (17)	0.13246 (10)	0.50681 (10)	0.0313 (3)	
H11A	1.289536	0.152865	0.469388	0.038*	
H11B	1.220667	0.170122	0.566350	0.038*	
C12	1.06567 (16)	0.13981 (9)	0.43879 (10)	0.0294 (3)	
H12A	1.049238	0.200354	0.417057	0.035*	
H12B	0.992111	0.124810	0.478761	0.035*	
C13	1.04263 (15)	0.08257 (9)	0.34476 (10)	0.0245 (3)	
C14	0.94946 (15)	0.10826 (9)	0.25786 (10)	0.0243 (3)	
C15	0.85684 (16)	0.18871 (9)	0.25623 (11)	0.0291 (3)	
H15A	0.897973	0.234904	0.220326	0.035*	
H15B	0.861504	0.208114	0.326504	0.035*	
C16	0.69784 (16)	0.17513 (10)	0.20514 (10)	0.0287 (3)	
H16A	0.649545	0.138771	0.248487	0.034*	
H16B	0.647285	0.231196	0.195821	0.034*	
C17	0.68889 (15)	0.13276 (8)	0.10503 (10)	0.0241 (3)	
C18	0.80528 (15)	0.08720 (8)	0.08480 (9)	0.0229 (3)	
C19	0.93568 (15)	0.06650 (8)	0.15694 (9)	0.0235 (3)	
C20	0.57464 (15)	0.12418 (8)	0.01881 (10)	0.0249 (3)	
C21	0.42983 (15)	0.15252 (9)	−0.00365 (11)	0.0286 (3)	
H21	0.392048	0.187645	0.042305	0.034*	
C22	0.34448 (16)	0.12776 (10)	−0.09437 (11)	0.0314 (3)	
H22	0.246121	0.145668	−0.110778	0.038*	
C23	0.40032 (16)	0.07647 (9)	−0.16307 (11)	0.0306 (3)	
H23	0.337837	0.060997	−0.224827	0.037*	
C24	0.54187 (16)	0.04754 (9)	−0.14491 (10)	0.0267 (3)	
C25	0.62890 (15)	0.07274 (8)	−0.05203 (10)	0.0239 (3)	
C26	0.59845 (17)	−0.01107 (10)	−0.21590 (11)	0.0325 (3)	
H26A	0.524816	−0.018001	−0.277929	0.049*	
H26B	0.621116	−0.067238	−0.183843	0.049*	
H26C	0.686416	0.013708	−0.232324	0.049*	
N1	1.10610 (13)	−0.06987 (7)	0.30159 (9)	0.0257 (3)	
N2	0.77040 (13)	0.05201 (7)	−0.01111 (8)	0.0237 (2)	
O1	1.02768 (11)	0.01812 (7)	0.13183 (7)	0.0289 (2)	
H1	1.0722 (19)	−0.0643 (11)	0.2358 (14)	0.035*	
H2	0.8269 (19)	0.0197 (12)	−0.0410 (13)	0.035*	

### (Z)-8-Methyl-2-(8-methyl-2,3,4,9-tetrahydrocarbazol-1-ylidene)-2,3,4,9-tetrahydrocarbazol-1-one Atomic displacement parameters (Å^2^)

**Table:**

	*U*^11^	*U*^22^	*U*^33^	*U*^12^	*U*^13^	*U*^23^
C1	0.0405 (9)	0.0315 (7)	0.0287 (7)	0.0002 (6)	0.0068 (6)	−0.0039 (6)
C2	0.0275 (7)	0.0305 (7)	0.0264 (6)	−0.0019 (6)	0.0097 (6)	0.0003 (5)
C3	0.0286 (7)	0.0320 (7)	0.0349 (7)	0.0026 (6)	0.0108 (6)	0.0014 (6)
C4	0.0268 (7)	0.0404 (8)	0.0343 (7)	0.0043 (6)	0.0042 (6)	0.0068 (6)
C5	0.0277 (7)	0.0406 (8)	0.0249 (6)	0.0002 (6)	0.0034 (6)	0.0032 (6)
C6	0.0239 (7)	0.0337 (7)	0.0242 (6)	−0.0014 (6)	0.0069 (5)	0.0008 (5)
C7	0.0242 (7)	0.0284 (7)	0.0234 (6)	−0.0014 (5)	0.0065 (5)	0.0016 (5)
C8	0.0248 (7)	0.0296 (7)	0.0212 (6)	−0.0030 (5)	0.0059 (5)	−0.0027 (5)
C9	0.0260 (7)	0.0334 (7)	0.0212 (6)	−0.0025 (6)	0.0054 (5)	−0.0014 (5)
C10	0.0344 (8)	0.0377 (8)	0.0224 (6)	−0.0026 (6)	0.0027 (6)	−0.0036 (6)
C11	0.0332 (8)	0.0352 (8)	0.0240 (6)	−0.0055 (6)	0.0025 (6)	−0.0066 (6)
C12	0.0311 (8)	0.0318 (7)	0.0248 (6)	−0.0011 (6)	0.0046 (6)	−0.0080 (5)
C13	0.0240 (7)	0.0273 (6)	0.0231 (6)	−0.0048 (5)	0.0069 (5)	−0.0046 (5)
C14	0.0233 (7)	0.0258 (6)	0.0247 (6)	−0.0016 (5)	0.0069 (5)	−0.0044 (5)
C15	0.0289 (7)	0.0292 (7)	0.0285 (7)	0.0022 (6)	0.0043 (6)	−0.0066 (5)
C16	0.0281 (7)	0.0321 (7)	0.0267 (6)	0.0030 (6)	0.0073 (6)	−0.0035 (5)
C17	0.0241 (7)	0.0241 (6)	0.0247 (6)	−0.0022 (5)	0.0065 (5)	0.0009 (5)
C18	0.0255 (7)	0.0222 (6)	0.0218 (6)	−0.0023 (5)	0.0066 (5)	−0.0005 (5)
C19	0.0246 (7)	0.0245 (6)	0.0219 (6)	−0.0023 (5)	0.0057 (5)	−0.0012 (5)
C20	0.0249 (7)	0.0243 (6)	0.0259 (6)	−0.0020 (5)	0.0061 (5)	0.0038 (5)
C21	0.0263 (7)	0.0290 (7)	0.0311 (7)	0.0007 (6)	0.0075 (6)	0.0052 (5)
C22	0.0250 (7)	0.0326 (7)	0.0354 (7)	0.0009 (6)	0.0030 (6)	0.0096 (6)
C23	0.0293 (8)	0.0315 (7)	0.0282 (7)	−0.0035 (6)	−0.0009 (6)	0.0068 (6)
C24	0.0287 (7)	0.0259 (6)	0.0244 (6)	−0.0022 (5)	0.0027 (5)	0.0051 (5)
C25	0.0245 (7)	0.0226 (6)	0.0244 (6)	−0.0029 (5)	0.0043 (5)	0.0042 (5)
C26	0.0314 (8)	0.0386 (8)	0.0259 (7)	−0.0045 (6)	0.0020 (6)	−0.0014 (6)
N1	0.0284 (6)	0.0268 (6)	0.0211 (5)	0.0000 (5)	0.0033 (5)	−0.0018 (4)
N2	0.0236 (6)	0.0253 (6)	0.0220 (5)	0.0005 (5)	0.0040 (4)	−0.0011 (4)
O1	0.0291 (5)	0.0344 (5)	0.0231 (5)	0.0059 (4)	0.0056 (4)	−0.0021 (4)

### (Z)-8-Methyl-2-(8-methyl-2,3,4,9-tetrahydrocarbazol-1-ylidene)-2,3,4,9-tetrahydrocarbazol-1-one Geometric parameters (Å, º)

**Table:**

C1—C2	1.500 (2)	C14—C19	1.4938 (18)
C1—H1A	0.9800	C14—C15	1.524 (2)
C1—H1B	0.9800	C15—C16	1.532 (2)
C1—H1C	0.9800	C15—H15A	0.9900
C2—C3	1.386 (2)	C15—H15B	0.9900
C2—C7	1.401 (2)	C16—C17	1.4934 (19)
C3—C4	1.411 (2)	C16—H16A	0.9900
C3—H3	0.9500	C16—H16B	0.9900
C4—C5	1.378 (2)	C17—C18	1.3796 (19)
C4—H4	0.9500	C17—C20	1.4235 (19)
C5—C6	1.410 (2)	C18—N2	1.3849 (17)
C5—H5	0.9500	C18—C19	1.4416 (19)
C6—C7	1.4158 (19)	C19—O1	1.2466 (17)
C6—C9	1.428 (2)	C20—C21	1.407 (2)
C7—N1	1.3741 (18)	C20—C25	1.422 (2)
C8—C9	1.3900 (19)	C21—C22	1.378 (2)
C8—N1	1.3946 (18)	C21—H21	0.9500
C8—C13	1.454 (2)	C22—C23	1.406 (2)
C9—C10	1.4955 (19)	C22—H22	0.9500
C10—C11	1.523 (2)	C23—C24	1.382 (2)
C10—H10A	0.9900	C23—H23	0.9500
C10—H10B	0.9900	C24—C25	1.4093 (19)
C11—C12	1.529 (2)	C24—C26	1.499 (2)
C11—H11A	0.9900	C25—N2	1.3742 (18)
C11—H11B	0.9900	C26—H26A	0.9800
C12—C13	1.5320 (18)	C26—H26B	0.9800
C12—H12A	0.9900	C26—H26C	0.9800
C12—H12B	0.9900	N1—H1	0.887 (18)
C13—C14	1.3759 (19)	N2—H2	0.887 (19)
			
C2—C1—H1A	109.5	C19—C14—C15	113.67 (11)
C2—C1—H1B	109.5	C14—C15—C16	113.45 (12)
H1A—C1—H1B	109.5	C14—C15—H15A	108.9
C2—C1—H1C	109.5	C16—C15—H15A	108.9
H1A—C1—H1C	109.5	C14—C15—H15B	108.9
H1B—C1—H1C	109.5	C16—C15—H15B	108.9
C3—C2—C7	116.00 (13)	H15A—C15—H15B	107.7
C3—C2—C1	122.95 (14)	C17—C16—C15	109.86 (12)
C7—C2—C1	121.04 (13)	C17—C16—H16A	109.7
C2—C3—C4	122.27 (14)	C15—C16—H16A	109.7
C2—C3—H3	118.9	C17—C16—H16B	109.7
C4—C3—H3	118.9	C15—C16—H16B	109.7
C5—C4—C3	121.19 (14)	H16A—C16—H16B	108.2
C5—C4—H4	119.4	C18—C17—C20	106.77 (12)
C3—C4—H4	119.4	C18—C17—C16	120.44 (12)
C4—C5—C6	118.45 (13)	C20—C17—C16	132.71 (13)
C4—C5—H5	120.8	C17—C18—N2	110.03 (12)
C6—C5—H5	120.8	C17—C18—C19	125.68 (12)
C5—C6—C7	119.05 (13)	N2—C18—C19	123.57 (12)
C5—C6—C9	134.37 (13)	O1—C19—C18	119.66 (12)
C7—C6—C9	106.53 (12)	O1—C19—C14	125.18 (12)
N1—C7—C2	128.55 (13)	C18—C19—C14	115.15 (12)
N1—C7—C6	108.41 (12)	C21—C20—C25	119.81 (12)
C2—C7—C6	123.03 (13)	C21—C20—C17	133.31 (13)
C9—C8—N1	108.34 (12)	C25—C20—C17	106.82 (12)
C9—C8—C13	124.95 (12)	C22—C21—C20	117.97 (14)
N1—C8—C13	126.54 (12)	C22—C21—H21	121.0
C8—C9—C6	107.64 (12)	C20—C21—H21	121.0
C8—C9—C10	123.76 (13)	C21—C22—C23	121.22 (14)
C6—C9—C10	128.58 (13)	C21—C22—H22	119.4
C9—C10—C11	108.92 (12)	C23—C22—H22	119.4
C9—C10—H10A	109.9	C24—C23—C22	123.03 (13)
C11—C10—H10A	109.9	C24—C23—H23	118.5
C9—C10—H10B	109.9	C22—C23—H23	118.5
C11—C10—H10B	109.9	C23—C24—C25	115.68 (13)
H10A—C10—H10B	108.3	C23—C24—C26	122.74 (13)
C10—C11—C12	112.42 (12)	C25—C24—C26	121.49 (13)
C10—C11—H11A	109.1	N2—C25—C24	129.40 (13)
C12—C11—H11A	109.1	N2—C25—C20	108.27 (12)
C10—C11—H11B	109.1	C24—C25—C20	122.28 (13)
C12—C11—H11B	109.1	C24—C26—H26A	109.5
H11A—C11—H11B	107.9	C24—C26—H26B	109.5
C11—C12—C13	114.43 (12)	H26A—C26—H26B	109.5
C11—C12—H12A	108.7	C24—C26—H26C	109.5
C13—C12—H12A	108.7	H26A—C26—H26C	109.5
C11—C12—H12B	108.7	H26B—C26—H26C	109.5
C13—C12—H12B	108.7	C7—N1—C8	108.88 (11)
H12A—C12—H12B	107.6	C7—N1—H1	125.7 (11)
C14—C13—C8	128.31 (12)	C8—N1—H1	120.3 (11)
C14—C13—C12	119.90 (13)	C25—N2—C18	108.08 (12)
C8—C13—C12	111.67 (11)	C25—N2—H2	124.9 (11)
C13—C14—C19	125.19 (12)	C18—N2—H2	127.0 (11)
C13—C14—C15	121.00 (12)		
			
C7—C2—C3—C4	−0.3 (2)	C15—C16—C17—C20	−162.64 (14)
C1—C2—C3—C4	178.38 (14)	C20—C17—C18—N2	1.35 (15)
C2—C3—C4—C5	−0.3 (2)	C16—C17—C18—N2	178.38 (12)
C3—C4—C5—C6	0.1 (2)	C20—C17—C18—C19	−169.14 (13)
C4—C5—C6—C7	0.7 (2)	C16—C17—C18—C19	7.9 (2)
C4—C5—C6—C9	−176.26 (16)	C17—C18—C19—O1	173.36 (13)
C3—C2—C7—N1	179.54 (14)	N2—C18—C19—O1	4.1 (2)
C1—C2—C7—N1	0.8 (2)	C17—C18—C19—C14	−7.94 (19)
C3—C2—C7—C6	1.2 (2)	N2—C18—C19—C14	−177.21 (12)
C1—C2—C7—C6	−177.52 (13)	C13—C14—C19—O1	−18.7 (2)
C5—C6—C7—N1	179.95 (13)	C15—C14—C19—O1	157.01 (13)
C9—C6—C7—N1	−2.32 (16)	C13—C14—C19—C18	162.71 (13)
C5—C6—C7—C2	−1.4 (2)	C15—C14—C19—C18	−21.60 (17)
C9—C6—C7—C2	176.29 (13)	C18—C17—C20—C21	176.85 (14)
N1—C8—C9—C6	3.10 (16)	C16—C17—C20—C21	0.3 (3)
C13—C8—C9—C6	178.54 (13)	C18—C17—C20—C25	−0.36 (15)
N1—C8—C9—C10	−175.53 (13)	C16—C17—C20—C25	−176.87 (14)
C13—C8—C9—C10	−0.1 (2)	C25—C20—C21—C22	0.95 (19)
C5—C6—C9—C8	176.72 (16)	C17—C20—C21—C22	−175.98 (14)
C7—C6—C9—C8	−0.51 (16)	C20—C21—C22—C23	−0.6 (2)
C5—C6—C9—C10	−4.7 (3)	C21—C22—C23—C24	0.2 (2)
C7—C6—C9—C10	178.05 (14)	C22—C23—C24—C25	0.0 (2)
C8—C9—C10—C11	−21.1 (2)	C22—C23—C24—C26	176.55 (13)
C6—C9—C10—C11	160.57 (15)	C23—C24—C25—N2	177.39 (13)
C9—C10—C11—C12	47.78 (17)	C26—C24—C25—N2	0.8 (2)
C10—C11—C12—C13	−57.26 (17)	C23—C24—C25—C20	0.35 (19)
C9—C8—C13—C14	178.14 (14)	C26—C24—C25—C20	−176.26 (12)
N1—C8—C13—C14	−7.3 (2)	C21—C20—C25—N2	−178.42 (12)
C9—C8—C13—C12	−5.8 (2)	C17—C20—C25—N2	−0.76 (15)
N1—C8—C13—C12	168.78 (13)	C21—C20—C25—C24	−0.8 (2)
C11—C12—C13—C14	−149.79 (14)	C17—C20—C25—C24	176.83 (12)
C11—C12—C13—C8	33.79 (17)	C2—C7—N1—C8	−174.22 (14)
C8—C13—C14—C19	−15.0 (2)	C6—C7—N1—C8	4.29 (16)
C12—C13—C14—C19	169.26 (13)	C9—C8—N1—C7	−4.62 (16)
C8—C13—C14—C15	169.63 (14)	C13—C8—N1—C7	−179.97 (13)
C12—C13—C14—C15	−6.1 (2)	C24—C25—N2—C18	−175.78 (13)
C13—C14—C15—C16	−132.98 (14)	C20—C25—N2—C18	1.59 (14)
C19—C14—C15—C16	51.13 (16)	C17—C18—N2—C25	−1.85 (15)
C14—C15—C16—C17	−49.66 (16)	C19—C18—N2—C25	168.88 (12)
C15—C16—C17—C18	21.23 (18)		

### (Z)-8-Methyl-2-(8-methyl-2,3,4,9-tetrahydrocarbazol-1-ylidene)-2,3,4,9-tetrahydrocarbazol-1-one Hydrogen-bond geometry (Å, º)

*Cg*1 and *Cg*3 are the centroids of the pyrrole (N1/C7/C6/C9/C8) and benzene (C2–C7) rings, respectively.

**Table:**

*D*—H···*A*	*D*—H	H···*A*	*D*···*A*	*D*—H···*A*
N1—H1···O1	0.887 (18)	1.887 (18)	2.6470 (16)	142.6 (15)
N2—H2···O1^i^	0.887 (19)	2.103 (19)	2.9574 (17)	161.4 (16)
C12—H12*B*···*Cg*1^ii^	0.99	2.90	3.876 (2)	170
C21—H21···*Cg*3^iii^	0.95	2.86	3.736 (2)	154
C26—H26*C*···*Cg*1^i^	0.98	2.70	3.549 (2)	145

Symmetry codes: (i) −*x*+2, −*y*, −*z*; (ii) −*x*+2, −*y*, −*z*+1; (iii) −*x*+3/2, *y*+1/2, −*z*+1/2.

## References

[bb1] Bruker (2002). *SMART*. Bruker AXS Inc., Madison, Wisconsin, USA.

[bb2] Bruker (2003). *SAINT-Plus*. Bruker AXS Inc., Madison, Wisconsin, USA.

[bb3] Cremer, D. & Pople, J. A. (1975). *J. Am. Chem. Soc.* **97**, 1354–1358.

[bb4] Farrugia, L. J. (2012). *J. Appl. Cryst.* **45**, 849–854.

[bb5] Groom, C. R., Bruno, I. J., Lightfoot, M. P. & Ward, S. C. (2016). *Acta Cryst.* B**72**, 171–179. 10.1107/S2052520616003954 PMC4822653 27048719

[bb6] Krause, L., Herbst-Irmer, R., Sheldrick, G. M. & Stalke, D. (2015). *J. Appl. Cryst.* **48**, 3–10. 10.1107/S1600576714022985 PMC4453166 26089746

[bb7] Matsuda, H., Hong, S. H., Ahn, S., Avena, R. F., Jeong, Y., Hwang, K. M., Son, E., Kang, S., Ko, S.-B., Kim, T. & Nakamura, M. (2025). *Commun. Mater.* **6**, 248.

[bb8] Sheldrick, G. M. (2008). *Acta Cryst.* A**64**, 112–122. 10.1107/S0108767307043930 18156677

[bb9] Sheldrick, G. M. (2015). *Acta Cryst.* C**71**, 3–8.

[bb10] Spackman, P. R., Turner, M. J., McKinnon, J. J., Wolff, S. K., Grimwood, D. J., Jayatilaka, D. & Spackman, M. A. (2021). *J. Appl. Cryst.* **54**, 1006–1011. 10.1107/S1600576721002910 PMC8202033 34188619

[bb11] Spek, A. L. (2020). *Acta Cryst.* E**76**, 1–11. 10.1107/S2056989019016244 PMC6944088 31921444

[bb12] Sridharan, M., Thiruvalluvar, A. A. & Rajesh, B. M. (2026). *Acta Cryst.* E**82**, 231–234. 10.1107/S2056989026000502 PMC12874232 41657517

[bb13] Westrip, S. P. (2010). *J. Appl. Cryst.* **43**, 920–925.

